# Sniffing Out the Structure of a Pheromone Receptor-Associated MHC Molecule

**DOI:** 10.1371/journal.pbio.0030280

**Published:** 2005-07-12

Although humans are thought to use their sense of smell to some extent when choosing a mate—or to a great extent, if perfume advertisements are to be believed—for many animals odor is centrally important in their mating decisions. Male moths, for example, are attracted over great distances to receptive mates by powerful species- and gender-specific cues known as pheromones.

Mammals, too, produce pheromones, although few mammalian pheromones have been unambiguously identified. These compounds have both long-lasting effects on the hormonal state of the animal receiving the signal and short-term effects on its social behavior. In rodents, pheromones are processed by a special part of the olfactory system called the vomeronasal organ (VNO), which lies between the nasal cavity and the top of the mouth.

In rats and mice, the VNO expresses two large families of genes encoding putative pheromone receptors—the V1Rs and the V2Rs. In 2003, it was discovered that for V2R receptors to be functional, they have to associate with members of the M10 and M1 families of non-classical major histocompatibility complex (MHC) class Ib molecules. Classical MHC class Ia molecules are a huge family of closely related, immunologically important molecules that present small pieces of foreign proteins (peptides) to T lymphocytes to help them recognize invading pathogens. By contrast, the smaller group of non-classical MHC molecules have both immune and non-immune functions. Intriguingly, peptides that bind to classical MHC class Ia molecules have been reported to activate V2R-expressing neurons. Furthermore, mice and rats mate preferentially with animals expressing MHC molecules different from their own.

Given all these pieces of information implicating both classical and non-classical MHC molecules in the social behavior of rodents, Pamela Bjorkman and her colleagues wanted to discover more about the relationship between these two classes of MHC molecules. To do this, they undertook a structural study of M10.5, one of the nine VNO-specific MHC class Ib proteins. They crystallized molecules of M10.5 expressed in insect cells and then used X ray crystallography to investigate how the M10.5 structure compares to that of classical MHC molecules. [Fig pbio-0030280-g001]

**Figure pbio-0030280-g001:**
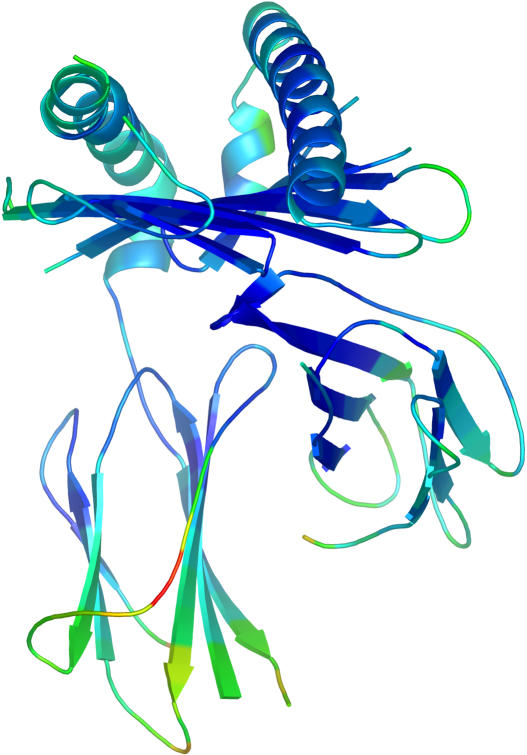
The structure of a pheromone receptor-associated molecule, shown here as a ribbon model, suggests that the molecule binds to an unknown compound

Overall, the structure turned out to be very similar but there was one big surprise. MHC class Ia molecules contain a characteristic open groove, which has thus far always been occupied by a peptide in crystal structures. The analogous open groove in the M10.5 was unexpectedly empty. However, experiments showed that the empty M10.5 molecule was thermally unstable, suggesting that the groove is normally occupied.

The researchers tried several approaches to identify the mysterious M10.5 ligand(s). First, they expressed M10.5 in mammalian cells rather than insect cells—insect cells lack the cellular machinery that normally loads peptides into MHC molecules—but still no peptides bound in the M10.5 groove. Then, they provided the M10.5 molecule with a mixture of peptides known to bind to MHC class I molecules. Again, no sign of peptide binding. Finally, the researchers used computer modeling to predict potential M10.5 groove occupants. From this analysis, they concluded that M10.5 and other M10s could bind a more restricted but longer set of peptides than MHC class Ia molecules. One possibility is that the M10.5 groove provides a binding site for V2Rs, but it might also bind pheromones.

Further experiments are now needed to identify the true binding partners of M10.5 and the other MHC class Ib molecules that are expressed in the VNO. Their eventual identification should provide insights into pheromone detection and facilitate the understanding of mating preferences in rodents. As for the mating preferences of humans, researchers will have to look elsewhere to solve that mystery since we do not appear to have M10 proteins, or a VNO!

